# Mr. Custance, on Singular Cases of Monstrosity

**Published:** 1799-07

**Authors:** George Custance

**Affiliations:** Kidderminster


### *To the Editors of the Medical and Physical Journal.*

Gentlemen,

The case of monstrosity given by Mr. Pulley, in your third Number, is extremely curious; and excites our admiration, that a child so formed, could "live about thirty hours", and was capable of taking a "little nourishment more than once". This surprising *lusus naturæ* is, however as to its formation, by no means a singular one. I remember seeing one very similar about twelve years ago. I think the subject was a boy; it moved its limbs when first born a few moments, but never breathed. The parietal bones stood up on each side, and being somewhat crooked, had the appearance of small horns. There was no brain, but a small excrescence, which seemed to form the beginning of the medulla oblongata, and entirely destitute of the occipital bone. I made a preparation of this subject, which remained with the gentleman with whom I then lived. Sometime in March last, I was called to the wife of a carpet-weaver, in this town, who was in labour. Upon *touching*, to ascertain the presentation, I had no doubt, from the soft sensation the part gave to my finger, that it was a breech case, and I fully expected a tedious labour; but, to my surprise, the very next pain protruded the *head*, and the whole body immediately followed. I now discovered that the *soft part* I had touched, was a little *nob* just above the occiput, which supplied the place of a proper brain.—There were no parietal bones, and so small a part of the frontal, that the eyes seemed to stand on the top of the forehead. The face was regularly formed, but the back part of the head was quite flat, in a line obliquely backward from the frontal sinuses to the first vertebræ of the neck. This monster was a girl, and quite motionless when born. I much wished to preserve it, but could not overcome the prejudices of the poor woman. Though I am quite sceptical as to the old doctrine, of external objects producing these monsters, yet I enquired of the mother, whether any thing had occurred during her pregnancy, to which she attributed the defect in the child's head. But she knew of nothing, excepting a fall, fall, about a fortnight before she was delivered, and even this was not much noticed at the time. All speculation about the *cause* of these *unnatural* appearances, seems premature, till "we know how the bones do grow in the womb of her that is with child," but we only know, that it is the "work of God, who maketh all."—If the above should be thought worthy a place in your very useful publication, I think the insertion of the following case, at the same time, may be of public utility, as it shews the extreme danger those unfortunate persons are always in, who labour under any species of hernia. On the 17th of this month, I was called to the wife of an alehouse-keeper, in this town, and found her in *articulo mortis*, as she expired in about half an hour after I saw her. Upon enquiring into the preceding circumstances, I learnt, that three days before, some persons had quarrelled in the house, and at last got to blows. The deceased had exerted herself in parting them, but in vain. From her fright, (as was supposed) she fainted, and had continued, at intervals, to do so till her death. Frequent vomitings came on, and she had had no stool from the first attack. It may appear extraordinary, under these circumstances, that she should not apply for medical assistance sooner; but all her symptoms, being attributed to a fright, created little alarm, either in her or her friends. This account did not satisfy me as to the cause of her death, and I suspected some blow had been given her in the quarrel. As her body appeared swelled, I gently pressed it with my hand, and in doing so, I felt through her cloaths a large substance, and, questioning her husband upon it, was informed she had had an umbilical rupture all her life-time, but had never found any inconvenience from it. I had now no doubt but this had done the mischief. A rumour was soon spread, that the poor woman lost her life from some person doing her violence in the scuffle; which, coming to the coroner's ear, he sent a message to Mr. Crane (surgeon, of this town) and me, desiring us to examine the body, and report our opinions to him, as to the cause of her death. We accordingly opened the umbilical tumor, containing some omentum, which was neither inflamed nor discoloured; but after carefully removing this, we found a small portion of intestine, not larger than a small walnut, completely strangulated, of a very dark brown colour. As no other marks of violence appeared about her, there could be no doubt as to the cause of her death. Her own exertion in the quarrel, probably protruded the gut, and inflammation and mortification ensuing, a very worthy woman was cut off from her husband and eight small children,

I am, Gentlemen,

Kidderminster, Your's,

*May* 24, 1799. GEORGE CUSTANCE.

